# Conformational plasticity of the intrinsically disordered protein ASR1 modulates its function as a drought stress-responsive gene

**DOI:** 10.1371/journal.pone.0202808

**Published:** 2018-08-23

**Authors:** Diana E. Wetzler, Federico Fuchs Wightman, Hernan A. Bucci, Jimena Rinaldi, Julio J. Caramelo, Norberto D. Iusem, Martiniano M. Ricardi

**Affiliations:** 1 Departamento de Química Biológica, Facultad de Ciencias Exactas y Naturales, Universidad de Buenos Aires, Buenos Aires, Argentina; 2 Instituto de Química Biológica de la Facultad de Ciencias Exactas y Naturales (IQUIBICEN), CONICET-Universidad de Buenos Aires, Buenos Aires, Argentina; 3 Departamento de Fisiología y Biología Molecular y Celular (FBMC), Facultad de Ciencias Exactas y Naturales, Universidad de Buenos Aires, Buenos Aires, Argentina; 4 Instituto de Fisiología, Biología Molecular y Neurociencias (IFIBYNE), CONICET-Universidad de Buenos Aires, Buenos Aires, Argentina; 5 Fundación Instituto Leloir, Buenos Aires, Argentina e Instituto de investigaciones Bioquímicas de Buenos Aires (IIBBA- CONICET), Buenos Aires, Argentina; Russian Academy of Medical Sciences, RUSSIAN FEDERATION

## Abstract

Plants in arid zones are constantly exposed to drought stress. The ASR protein family (Abscisic, Stress, Ripening) -a subgroup of the late embryogenesis abundant superfamily- is involved in the water stress response and adaptation to dry environments. Tomato ASR1, as well as other members of this family, is an intrinsically disordered protein (IDP) that functions as a transcription factor and a chaperone. Here we employed different biophysical techniques to perform a deep *in vitro* characterization of ASR1 as an IDP and showed how both environmental factors and *in vivo* targets modulate its folding. We report that ASR1 adopts different conformations such as α-helix or polyproline type II in response to environmental changes. Low temperatures and low pH promote the polyproline type II conformation (PII). While NaCl increases PII content and slightly destabilizes α-helix conformation, PEG and glycerol have an important stabilizing effect of α-helix conformation. The binding of Zn^2+^in the low micromolar range promotes α-helix folding, while extra Zn^2+^ results in homo-dimerization. The ASR1-DNA binding is sequence specific and dependent on Zn^2+^. ASR1 chaperone activity does not change upon the structure induction triggered by the addition of Zn^2+^. Furthermore, trehalose, which has no effect on the ASR1 structure by itself, showed a synergistic effect on the ASR1-driven heat shock protection towards the reporter enzyme citrate synthase (CS). These observations prompted the development of a FRET reporter to sense ASR1 folding *in vivo*. Its performance was confirmed in *Escherichia coli* under saline and osmotic stress conditions, representing a promising probe to be used in plant cells. Overall, this work supports the notion that ASR1 plasticity is a key feature that facilitates its response to drought stress and its interaction with specific targets.

## Introduction

Plants in arid zones are constantly exposed to drought stress and respond by adopting different survival strategies [1]. The accumulation of proteins of the late embryogenesis abundant (LEA) superfamily constitutes one of the most conserved responses to that stressful condition [2]. LEA proteins are classified into different groups based on amino acid sequence motifs [3]. Among them, the ASR (Abscisic, Stress, Ripening) protein family was first described in tomato [4] and is involved in water stress response and adaptation to dry environments [5]. ASR is widespread in the plant kingdom including numerous relevant crops, though it is absent in Arabidopsis [6]. Tomato ASR1 is a small protein and, like others members of the LEA superfamily, it has a high content of small and charged amino acids [4]. It consists of two domains: a DNA-binding domain located at the C-terminus and an N-terminal domain postulated as a Zinc-binding domain [7]. In addition, it has been found that it can form homodimers [8][9][10][11].

Similarly to other members of this family, ASR1 was predicted to be an IDP [7]. Many examples in the literature clearly illustrate the functional relevance of IDPs in central areas of plant biology, including abiotic stress and transcriptional regulation [12]. Various roles have been proposed for ASR proteins during abiotic stress. *In vitro* experiments suggested that tomato ASR1 can stabilize a number of proteins against denaturation suggesting a chaperone function under drought stress [13]. Other authors speculated that these proteins could buffer high Zn^2+^ concentrations that may accumulate in the cytoplasm under abiotic stress, giving time for cation-efflux transporters to restore the Zn^2+^ balance. On the other hand, a genome-wide approach showed that tomato ASR1 is a transcription factor in tomato stressed leaves [14]. Similarly, the role as a transcription factor of the orthologous rice ASR5 during aluminum-induced stress has been highlighted [15]. Although several molecular functions for ASR1 have been suggested, there are still many questions about their relationship with its IDP nature and how environmental factors (temperature, pH, ion concentration) finely tune ASR1 function under abiotic stress.

We present here an exhaustive biophysical characterization and show how different environmental conditions favour various conformations of ASR1 like polyproline type II structure or α-helix. Our data reveal that ASR1-binding of Zn^2+^ promotes its partial folding as an α-helix monomer, a prerequisite to bind its specific DNA sequence. In addition, higher Zn^2+^ concentrations induce ASR1 dimerization. These results support the idea that folding behavior determines ASR1 function as chaperone and/or transcription factor. Finally, in order to visualize its folding *in vivo*, we designed a FRET construct based on ASR1. This sensor responds to saline and osmotic stress when expressed in bacterial cells, representing a promising tool to understand the water deficit-stress response in plant cells.

## Materials and methods

### Cloning

Cloning was performed by standard restriction-ligation protocols. DNA inserts were sequentially cloned into the pQE32 plasmid. Inserts were generated by polymerase chain reaction (PCR) adding different flanking restriction sites. As templates for PCR reactions, we used peCALWY-4 (a gift from Maarten Merkx, Addgene plasmid # 22236) for amplification of sticky citrine (stCit) and sticky ECFP (stECFP) and pet28CLY-6 (a gift from Maarten Merkx, Addgene plasmid # 21765) for ECFP and EYFP. stCit and EYFP were amplified with primers by adding BamHI and SacI sites (Fw-BamHI CATGGGATCCCGCATGGTGGCAAGGGCGAG, Rev-SacI TCCGAGCTCGCTTGTACAGCTCGTCCAT), ASR1 with primers adding SacI and XmaI sites (Fw-SacI AGCGAGCTCGGATGGAGGAGGAGAAACAC, Rev-XmaI CATCCCGGGGAAGAGATGGTGGTGTCC) and stECFP/ECFP with primers adding XmaI and HindIII sites (Fw-XmaI TTCCCCGGGATGGTGAGCAGGGCGAG, Rev-HindIII CTTAAGCTTGCTTGTACAGCTCGTCCAT). The original HISx6 tag was removed by amplification with primers Fw CACCATCACCATCACCATGG and Rev-BamHI CCCGGATCCTCTCATAGTTAATTT, and subsequent restriction with BamHI and religation.

### Buffers

Buffer P: 100 mM NaCl, 20 mM sodium phosphate pH 7.5,

Buffer T: 100 mM NaCl, 20 mM tris-HCl 2 0mM pH 7.5,

Buffer H: 100 mM NaCl, 20 mM HEPES 20 mM pH 7.4,

For the ionic strength titration, 0 to 1 M NaCl

Buffer C-T: 100 mM NaCl, 50 mM final citrate-phosphate on different ratios to obtain pH values of 2.0, 3.0, 4.0, 5.0, 6.0, 7.0, and 8.0

Buffer S75: sodium phosphate 20mM pH 7.5, 500 mM NaCl

### Protein purification

Transformed *Escherichia coli* M15 strains harboring plasmids for the expression of the different constructs were grown over night at 37°C with agitation (180 rpm). Large-scale cultures were started at an initial OD_600_ of 0.5. For each construct, we used a total volume of 1 L of LB media distributed in two baffled 2-liter Erlenmeyer flasks. After a one-hour incubation, cultures were induced with 0.1 mM IPTG harvested by centrifugation after 3 hours. Protein was then purified using Ni^2+-^ affinity chromatography (HisTrap column). Briefly, pelleted cells were resuspended in a binding buffer containing 100 mM imidazole, treated with lysozyme for 30 min at 37°C and then sonicated. The lysate was cleared by centrifugation, filtered and loaded into the HisTrap column following the manufacturer´s protocol. After elution, samples were dialyzed in Buffer S75. Untagged ASR1 protein was further purified by gel filtration chromatography on semi-preparative a Superdex S75 column (GE Healthcare). Peaks corresponding to the expected full-length protein were pooled and concentrated by centrifugation in Amicon Ultra-4 devices (Millipore) with a 10-kDa cutoff. Purified proteins were analyzed by SDS-PAGE. ASR1 showed a single band corresponding to the expected MW. At high protein concentrations, the ASR1 dimer was observed as well. ASR1-based sticky and non-sticky sensors showed a single band while minor contaminants were observed for ASR1-eCFP, ASR1-cYFPand CLY-6 constructs (S1 Fig).

### Size exclusion chromatography (SEC)

A Superdex S75 column was used for SEC analysis. The column was equilibrated in Buffer S75 with or without the specified Zn^2+^ concentrations. Injections were performed with 63 μg of ASR1 recombinant protein loaded in 100 μL of the same buffer. A 0.8 mL/min flow was used and chromatograms monitoring absorbance at 280 nm were obtained.

### Dynamic light scattering (DLS)

DLS measurements were carried out on a Zetasizer Nano S DLS device (Malvern Instruments). ASR1 concentration was kept at 45 μM in order to stand above the instrument detection limit (minimum concentration recommended is 0.5 mg/L for particle size <10 nm). Measurements were performed in Buffer T and incubated with different amounts of ZnCl_2_. The temperature was maintained at 25°C by a Peltier control system. Results were processed employing the software package included in the equipment.

### Static light scattering (SLS)

The average molecular weight was determined by SLS using a PD2010 light scattering instrument (Precision Detectors Inc.) connected in tandem to a high-performance liquid chromatography system and an LKB 2142 differential refractometer. The 90° light scattering and refractive index signals of the eluting material were analyzed with Discovery32 software supplied by Precision Detectors Inc. SEC runs were carried out on an analytic Superdex 75 column. The protein concentration used was 3 mg/mL -The elution. Elution was performed in buffer S75 with or without the specified Zn^2+^ concentrations.

### Circular dichroism spectroscopy (CD)

CD measurements were carried out with a Jasco J-815 spectropolarimeter. CD spectra in the far UV region were collected using a Peltier temperature-controlled sample holder in a 0.1 cm-path cell. Isothermal wavelength spectra were achieved at 25°C with a scan speed of 100 nm/min, a response time of 4 sec and averaged over at least 10 scans. Variable temperature spectra were acquired every 5 degrees between 5°C and 90°C. Mean Residual Molar Ellipticity ([θ]_MRW_ (deg cm^2^dmol^-1^res^-1^) was calculated from measured ellipticity θ (mdeg) values. The percentage of amino acids in α-helical conformation was estimated by using an empirical equation where the mean residual molar ellipticity at 222 nm (${\left[\theta \right]}_{MRW}^{222\;nm}$) is related to the number of amino acids (*n*) in α-helical conformation [16].

$${\left[\theta \right]}_{MRW}^{222\;nm}=-39500\;\left(1-\frac{2.57}{n}\right)$$ (1)

We used increasing amounts of trifluoroethanol (TFE) and guanidinium chloride (GdnCl) to stabilize α-helix and PII conformations, respectively. Assuming that populations of polyproline type II, PII (for the TFE titration) and α-helix (for the GdnCl titration) do not change significantly during titration, it is possible to fit the observed ellipticity into a two-state equilibrium model [17][18]).

initial↔α−helixorinitial↔PII (2)

We considered the free energy (ΔG) for α-helix formation to depend linearly on the TFE/water molar ratio and the PII structures to depend linearly on the molar concentration of GdnCl, with a proportionality constant m:

$${\Delta G}_{intial↔\alpha -helix}^{TFE}={\Delta G}_{initial↔\alpha -helix}^{water}-m_{\alpha -helix}\left(\frac{\left[TFE\right]}{\left[water\right]}\right)$$ (3.A)

$${\Delta G}_{initial↔\alpha -helix}^{\left(GdnCl\right)}={\Delta G}_{initial↔\alpha -helix}^{water}-m_{PII}[GdnCl]$$ (3.B)

The mean residual molar ellipticity at a fixed wavelength, λ (222 nm for α-helix or 218 nm for PII) during the titration can be fitted to the following equation to extract values for ΔG and m:

$${\left[\theta \right]}_{MRW}^{\lambda }=\frac{{\left[\theta \right]}_{MRW}^{X}+{\left[\theta \right]}_{MRW}^{water}exp(-{\Delta G}^{x}/RT)}{1+{\left[\theta \right]}_{MRW}^{water}exp(-{\Delta G}^{x}/RT))}$$ (4)

where ${\left[\theta \right]}_{MRW}^{water}$ and ${\left[\theta \right]}_{MRW}^{x}$ are the mean residual molar ellipticity without co-solvent and with saturating co-solvent concentrations, respectively, R is the gas constant, T is the temperature and ΔG ^x^ is the free energy at each co-solvent concentration (from Eq 3A or 3B)

### dsDNA oligonucleotide probe assembly

100 μM of fluorescein (FITC) 5´-modified ssDNA was incubated with a 150 μM of its complementary non-modified ssDNA in Buffer H. Oligonucleotides containing ASR1 consensus sequence TATTGGGCTTGG (oligoC) and scrambled sequence with the same nucleotide content AGTTCTGGTTGG (oligoS) were incubated at 75°C for 5 min and then cooled down to 10°C with a slow rate (10°C/min). Annealed products (FITC-oligoC and FITC-oligoS) were analyzed on an agarose gel and stained with ethidium bromide to check the presence of the dsDNA molecule. As negative controls, single strand oligonucleotides were also run. Unlabeled dsDNA oligoC was also performed in the same way.

### Fluorescence anisotropy and fluorescence intensity DNA titrations

Anisotropy measurements were performed with a PTI Quanta-Master QM4 fluorometer (Horiba) equipped with polarizers. 250 nM FITC-labeled dsDNA was incubated in buffer H with 100 μM ZnCl_2_. Titrations were performed by the addition of increasing amounts of ASR1 protein. FITC was excited at 495 nm (4 nm bandwidth) and emission collected at 515 nm (2 nm bandwidth). Signals were averaged for 60 seconds. To determine the stoichiometry, titrations of DNA fluorescence were performed with the same fluorometer, keeping the FITC oligonucleotide concentration at 1250 nM.

### Thermal CS aggregation assay

Pig heart CS (100 nM final concentration) was added to a 43°C-preheated solution containing varying amounts of ASR1 and/or 100 mM of trehalose in Buffer T with 150 mM NaCl with or without 50 μM ZnCl_2_. A control experiment with ASR1 1 μM without CS was performed in parallel. Aggregation events were followed by monitoring light scattering at 360 nm, using the absorbance mode, in a multi-well spectrophotometer (FLUOstar OPTIMA). All experiments were performed at least three times.

### *In vitro* FRET measurements

Fluorescence was measured in a fluorometer (Aminco Bowman Series 2). We employed 1μM final concentration of protein incubated in buffer H. Zinc titrations were performed by adding different amounts of ZnCl_2_ in the same buffer. Emission spectra were collected using a 420 nm excitation with a 4-nm bandwidth. FRET changes were followed by the ratio between the donor maximum emission and the emission at the isosbestic wavelength (I_donor_/I_Isosbestic_) as recently described for this type of molecules [19].

### *In vivo* FRET assays

*Escherichia coli* M15 strains harboring plasmids for the expression of the different constructs were grown overnight at 37°C with 180 rpm shaking in M9 media supplemented with 1μg/mL thiamine and biotin and 0.5% glycerol as a carbon source. The following day, cultures were diluted in fresh M9 media to an OD_600_ = 0.25. After reaching an OD_600_ = 0.8, cultures were induced with 40 μM (final concentration) IPTG. After 14 hours, cells were mixed with increasing amounts of NaCl, homogenized and immediately measured in the fluorometer at a final OD_600_ of 0.5. Both I_aceptor_/I_Isosbestic_ or I_donor_/I_Isosbestic_ were measured in the same way as in the *in vitro* experiments.

### Binding models

Dissociations constants for ASR1-oligo ($K_{d}^{oligo}$) or ASR1-Zn^2+^ ($K_{d}^{{Zn}^{2+}}$) complexes were obtained from the 1:1 stoichiometry binding model by fitting the following equation:

$$y=y_{0}+\frac{\left(y_{f}-y_{0}\right)}{2\;{\left[ASR1\right]}_{T}}\{(K_{d}^{x}+{\left[ASR1\right]}_{T}+{\left[x\right]}_{T})+\sqrt{{\left(K_{d}^{x}+{\left[ASR1\right]}_{T}+{\left[x\right]}_{T}\right)}^{2}-4{\left[ASR1\right]}_{T}\;{\left[x\right]}_{T}}\}$$ (5)

where:

*y* is the molecular property being monitored (anisotropy, mean molar residual ellipticity or I_donor_/i_sosbestic_ in each case), *y*_*0*_ and *y*_*f*_ are the properties for the free receptor and the complex, respectively, [ASR1]_T_ is the total protein concentration and [x]_T_ is the total oligonucleotide or Zn^2+^ concentration in each case.

The dimer formation constant in the presence of higher Zn^2+^ concentration, K _dim_, was estimated by postulating the formation of a ternary complex after the formation of the ASR1-Zn^2+^ binary complex:

$$2\;ASR1-{Zn}^{2+}+{Zn}^{2+}↔{Zn}^{2+}{-(ASR1-{Zn}^{2+})}_{2}$$ (6)

Considering the presence of free ASR1 negligible in the second event observed in FRET binding experiments, we obtained the following equation for the proposed model:

$$y=y_{0}+\frac{\left(y_{f}-y_{0}\right)}{2\;{\left[ASR1\right]}_{T}}\left\{{\left(4[ASR1\right]}_{T}+\frac{1}{K_{dim}[{Zn}^{2+}]})+\sqrt{{\left(4{\left[ASR1\right]}_{T}+\frac{1}{K_{dim}[{Zn}^{2+}]}\right)}^{2}-{\left(4{\left[ASR1\right)}_{T}\right)}^{2}}\right\}$$ (7)

where:

*y* is I_donor_/I_Isosbestic_, *y*_*0*_ and *y*_*f*_ are the ratio for the ASR1-Zn^2+^and Zn^2+^-(ASR1-Zn^2+^)_2_ complexes, respectively, [ASR1]_T_ is the total protein concentration and [Zn^2+^] is the free Zn^2+^ concentration.

The assumption that free ASR1 was negligible is reasonable under the experimental conditions: the low concentration of ASR1 employed (1 μM), the higher amounts of Zn^2+^added and the μM-range dissociation constant obtained for the ASR1-Zn^2+^ complex. Under these experimental conditions, the free Zn^2+^ concentration can be considered equal to the total Zn^2+^ concentration.

## Results

### ASR1 plasticity

As an IDP, ASR1 is expected to display an extended and dynamic secondary structure that can be stabilized by changes in the chemical environment. Therefore, we assayed the effect of different agents on its conformation. To this end, we explored ASR1 structural changes upon the addition of both TFE, widely used to induce α-helical formation, and GdnCl, which has a stabilization effect of PII structure on IDPs [20,21].

**Fig 1:**
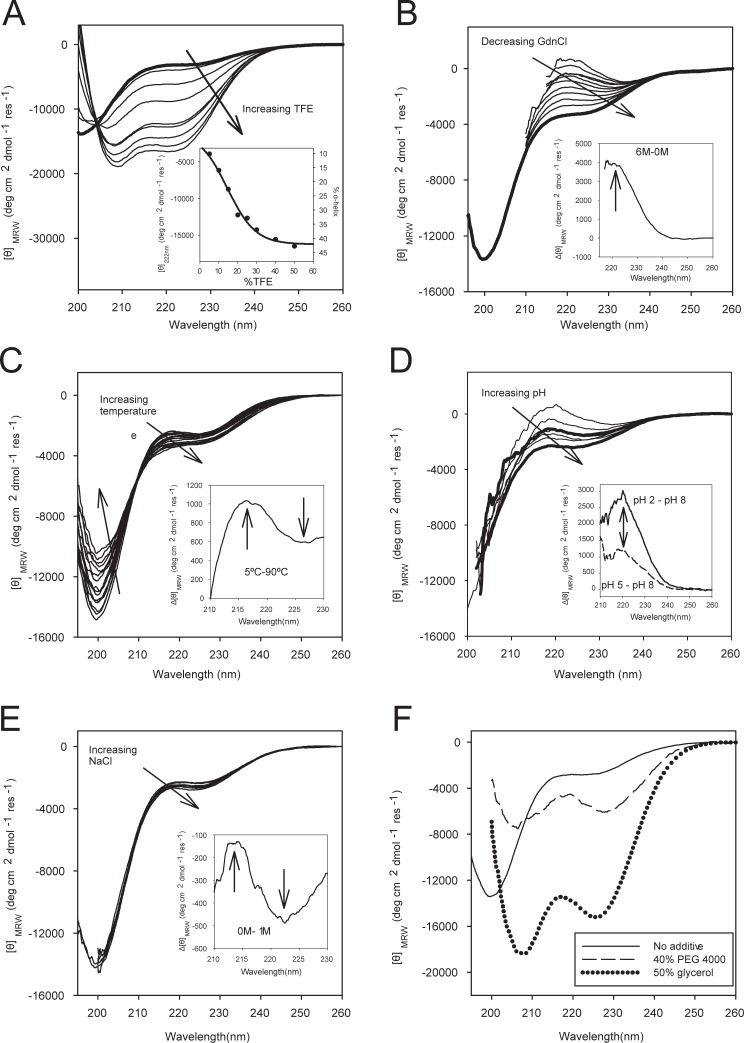
ASR1 secondary structure in different environments. CD spectra of ASR1 (10 μM). A) At different TFE concentrations from 0 (thick line) to 50%, inset: Mean Residual Molar Ellipticity at 222 and % of calculated α-helix as a function of TFE concentration. The solid line corresponds to the fit of 1:1 binding model (Eq 4) B) At different GdnCl concentrations (0 M in thick line); inset, differential spectrum between 6 M and 0 M. C) At increasing temperatures (from 5 to 90°C); inset, differential spectrum between 5°C and 90°C. D) At pH 2, 3, 4, 5, 6, 7 and 8; inset, differential spectrum between pH 5 and pH 8. E) At increasing amounts of NaCl; inset, difference spectrum between 1 M and 0 M. F) Without any stabilizing or denaturant agents (full line) in the presence of crowding agents: 40% of PEG 4000 (dash line) or 50% glycerol (dotted line). More noticeable spectral changes are shown in arrowheads in the inset figures.

The CD spectrum of ASR1, without any additive, displayed a minimum dichroic peak centered at 200 nm, typical of disordered conformations, and a low signal above 205 nm, characteristic of a more ordered structure (Fig 1A). These observations are in accordance with *in silico* predictions and experimental observations describing ASR1 as a disordered protein [8]. Taking into account the signal at 222 nm (Fig 1A), we estimated an 8% of α-helical content. Interestingly, the addition of TFE at a low concentration was sufficient to stabilize α-helix conformation, with characteristic negative bands at 208 and 222 nm (Fig 1A) whereas 10% TFE was enough to stabilize 16% of the amino acids in α-helical conformation, reaching a maximum of 43% α-helical content. The spectra showed a unique isodichroic point, at 205 nm, typical for a two-state transition (Fig 1A), which was found to be cooperative (Fig 1A, inset). A free energy value for the α-helix structure formation (${\Delta G}_{initial↔\alpha -helix}^{water}$) of (1.1± 0.4) kcal/mol was calculated considering a two-state model (see Materials and Methods). On the other hand, the presence of GdnCl produced an increase in the band at 218 nm in the CD spectra, indicative of stabilization of the PII structure (Fig 1B and inset) [22]. In this case, the low signal-to-noise ratio due to the small spectral change and the presence of the denaturant introduces a large error. However, we were able to estimate a free energy for PII structure formation ${\Delta G}_{initial↔PII}^{water}$ of ~ 0.6 kcal/mol. This evidence supports the fact that ASR1 is an IDP that can easily adopt different conformations such as α-helix or PII.

### External factors that stabilize ASR1 different conformations

#### Low temperatures and low pHs

As expected, the PII configuration was stabilized at low temperatures [23]. ASR1 displayed maximum PII configuration at 5°C and minimum PII stabilization at 90°C. The difference CD spectrum uncovered spectral features, namely the typical broad positive band at 218 nm and a negative band a 228 nm (Fig 1C). The CD spectra also presented an isodichroic point at 209 nm (Fig 1C and inset). A careful analysis of the temperature effect suggested that higher temperatures **slightly** stabilized the α-helix conformation. This effect has been previously observed for other IDPs [24] and could be due to the increase of hydrophobic attractive interactions at higher temperatures that promote folding.

PII conformation is also known to undergo a pH-induced transition [25]. In the case of ASR1, decreasing pH values in the range of physiological ones (from 8 to 5) [26] were able to stabilize the PII conformation. This effect is clearly appreciated in the difference spectrum between pH 5.0 and pH 8.0 and even more noticeable at lower pH values as can be seen at pH 2.0 (Fig 1D and inset). These results show that ASR1 would be partially folded in PII conformation at low temperatures and low pHs.

#### Macromolecular crowding agents and ionic strength

Next, we studied the effect of ionic strength and molecular crowding agents that mimic the cell interior under drought stress on ASR1 folding. While increasing concentrations of NaCl from 0 to 1 M increased PII conformation and slightly destabilized α-helix conformation (Fig 1E and inset), the addition of glycerol or crowding agents like polyethylene glycol (PEG) clearly stabilized an α-helix conformation (Fig 1F). Particularly, 50% of glycerol stabilized 37% of ASR1 amino acids in α-helix whereas 40% of PEG stabilized 14% of them. The more pronounced effect of glycerol may be attributed to a similar behavior to TFE and other alcohols on stabilization α-helix conformations[27].

#### Effect of Zn^2+^

**Fig 2:**
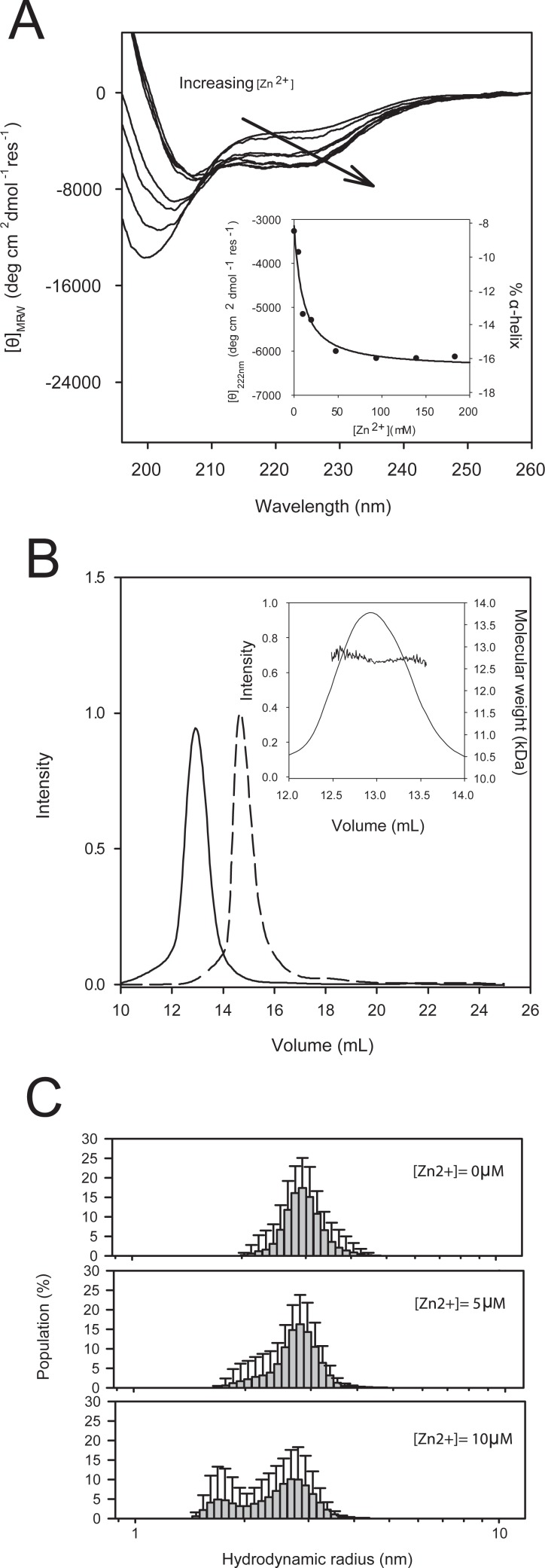
Zinc-mediated ASR1 folding. A) CD spectrum changes for ASR1 (2 μM) after the addition of increasing amounts of ZnCl_2_; inset: Mean Residual Molar Ellipticity at 222 nm and % of calculated α-helix as a function of Zn^2+^ concentration. The solid line corresponds to the fit of 1:1 binding model (Eq 5). B) Elution profile of ASR1 (63 μg) by SEC using an analytical Superdex 75 column: without Zn^2+^ (full line) and with 10 μM Zn^2+^ (dash line); inset: Molar weight determination by SLS for the protein without Zn^2+^ addition. C) DLS measurements of 40 μM ASR1 with 0, 5 and 10 μM Zn^2+^. Frequency histogram of hydrodynamic radius (logarithmic scale) is plotted with the corresponding standard deviation.

As Zn^**2*+***^ is an important ASR1 cofactor [7], we assessed the effect of this cation on ASR1 folding and quaternary structure. Changes in the secondary structure were followed by CD during Zn^2+^ titrations of a 2 **μ**M ASR1 solution. In the absence of Zn^2+^, the ASR1 CD spectrum displayed the same IDP-like spectral features. However, increasing concentrations of Zn^2+^induced a shift on the negative band from 200 nm to 208 nm and the appearance of the 222 nm negative band, distinctive of an α-helix conformation. The resulting spectrum showed an isodichroic point at 205 nm and presented no changes above 50 **μ**M ZnCl_2_ (Fig 2A and inset). At this point, the protein had 16% of its amino acids in α-helix conformation. Consistently, the addition of EDTA restored the initial spectrum (S2 Fig). Following the CD signal change at 222 nm in the Zn^2+^ titration experiment and considering a 1:1 stoichiometry (see Materials and Methods), we estimated an equilibrium dissociation constant between ASR1 and Zn^2+^, $K_{d}^{{Zn}^{2+}}$, of (9 ± 2) **μ**M. On the other hand, SEC performed on a Superdex S75 column showed that ASR1 eluted as a single peak. SLS measurements of this peak indicated an average molecular weight of (12.7 ± 0.1) kDa (Fig 2B). When SEC was carried out in presence of Zn^2+^, ASR1 displayed a smaller hydrodynamic radius while keeping a similar molecular weight, suggesting that the protein molecules remained monomeric. ASR1 hydrodynamic behavior was confirmed by DLS. Particularly, the hydrodynamic radius of (2.97 ± 0.25) nm estimated in the absence of Zn^2+^ was in concordance with the 2.95 nm value predicted for a 12.7 kDa IDP monomer [28]. The hydrodynamic radius distribution after the addition of 5 **μ**M Zn^2+^ revealed the appearance of a population of smaller size. These two populations were more evident at10 **μ**M Zn^2+^. The first population exhibited a radius similar to the observed one in the absence of Zn^2+^ (2.72 ± 0.18) nm while the second one presented an average hydrodynamic radius of (1.81 ± 0.05) nm (Fig 2C).

### Zn^2+^ effects on ASR1 functions

#### DNA binding

**Fig 3:**
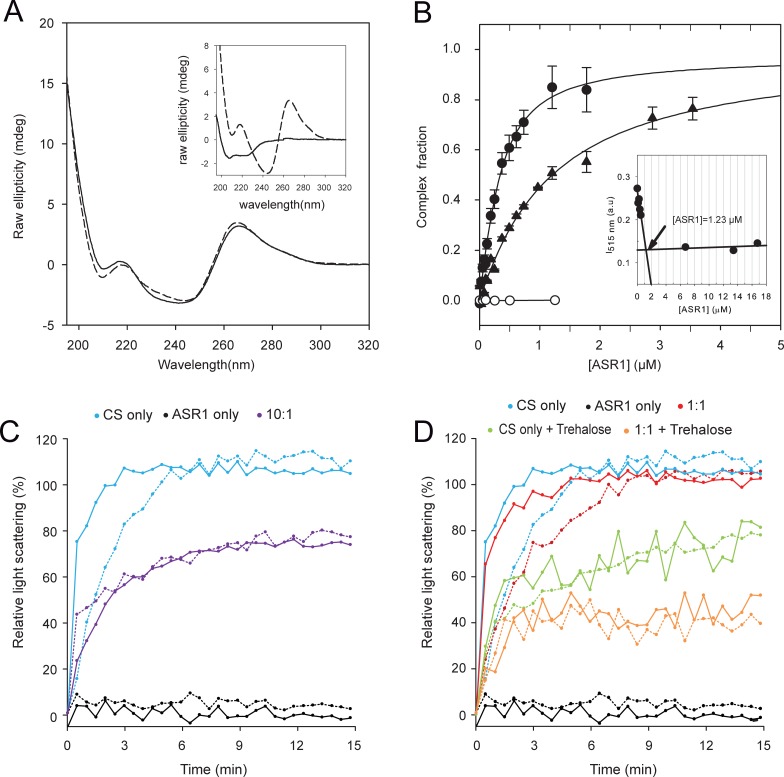
Zn^2+^ effects on ASR1 functions. A). CD spectrum at 25°C of ASR1 (2 μM) with oligoC (15 μM) full line corresponds to the oligo-protein mixture and dash line represents the algebraic sum of the individual spectrum; inset: spectra of the ASR1 (full line) and oligoC (dash line) alone. B) Fluorescence anisotropy titration. Binding experiments were performed by adding recombinant ASR1 protein to a fixed amount of FITC dsDNA (250 nM). Measurements performed in buffer H with Zn^2+^ (100 μM): FITC-oligoC (full circles) and FITC-oligoS (full triangles) and without Zn^2+^: FITC-oligoC (empty circles). Lines correspond to the best fit to the Eq 5 and error bars correspond to the standard errors of two individual experiments. Inset: Fluorescence intensity change as a function of ASR1 concentration performed on nearly saturating conditions (FITC-oligoC: 1250 nM). The arrow indicates the 1:1 stoichiometry. C and D) CS (100 nM) was incubated at 43°C at the indicated ASR1:CS ratios in absence (dashed line) or presence (full line) of 50 μM Zn ^2+^. +Trehalose indicates the addition of 100 mM trehalose. Plots show light scattering (OD_360nm_) relative to the highest value obtained on the CS alone sample. CS alone and ASR1 alone were used as controls. Mean values of at least 3 measurements.

Given that ASR1 can interact with DNA containing the specific sequence TGGGCT *in vivo* [14], we tested if this interaction could be also detected in solution by CD spectroscopy. For this purpose, a 10 bp oligonucleotide encompassing the 6-bp consensus sequence (oligoC) was employed. The CD spectrum of a mix of ASR1 (2 **μ**M) with an excess of oligoC (15 **μ**M), previously incubated for 15 minutes in a buffer containing 100 **μ**M of ZnCl_2_, presented slight differences with the algebraic sum of the spectra derived from protein and oligonucleotide separately. Interestingly, a modest increment and a small shift in the oligo signal was perceived at 260 nm, where there is not protein contribution, suggesting that the oligoC suffered some minor rearrangement after binding to ASR1. Additionally, there was a difference between the experimental mix spectrum and the algebraic sum spectrum in the 200–260 nm range, which confirms the interaction (Fig 3A and inset). As both ASR1 and oligoC show a dichroic signal in this region, it was not possible to discriminate if this change reflects structural changes in the oligonucleotide, in the protein or in both.

To further characterize the interaction of ASR1 with DNA, we used 5´ FITC modified oligonucleotides and followed the changes in fluorescence anisotropy or FITC fluorescence after the addition of ASR1. Titration experiments in the presence of Zn^2+^ showed that anisotropy increased until reaching a plateau, whereas no change was observed in the absence of Zn^2+^ (Fig 3B). To determine the binding stoichiometry, we performed assays at a higher FITC-oligoC concentration and followed FITC fluorescence changes, yielding a 1:1 value (Fig 3B inset). Dissociations constants for the ASR1-oligoC ($K_{d}^{oligoC}$) complex were obtained from the 1:1 stoichiometry binding model by fitting anisotropy changes to Eq 5 (see Materials and Methods). We obtained a $K_{d}^{oligoC}$ of (216 ± 10) nM. To investigate the sequence-specificity of ASR1 binding, we employed another oligonucleotide with the same base content but in a scrambled sequence order (FITC-oligoS). The latter presented a lower affinity, with a $K_{d}^{oligoS}$ of (1078 ± 46) nM. (Fig 3B). In summary, we validated that the DNA binding is Zinc- dependent and that ASR1 displays a 5-fold preference towards its specific DNA target.

#### ASR1 chaperone activity

ASR1 chaperone activity was previously observed at relatively high concentrations of ASR1 using the CS thermal aggregation assay [13]. The presence of chaperones and other protective molecules, can prevented heat induced CS denaturing and its subsequent aggregation. Following CS aggregates, through light scattering, represent an easy way to asses molecular chaperone activity [29]. Considering that Zn^2+^ induced alpha helix configuration of ASR1, we wanted to evaluate if this structuring can modify its chaperone activity. To assess this, we first performed CS thermal aggregation assays in the presence and absence of Zn^2+^. Our first assay confirmed that ASR1 protects CS from aggregation (10:1 vs CS alone) and that ASR1 protein alone does not form aggregates that contribute to the scattering signal (Fig 3C). Addition of Zn^2+^ accelerated the aggregation of CS, but did not influence ASR1 chaperone activity (Fig 3C). We also examined whether Zn^2+^ is able to modify ASR1 chaperone activity in the presence of the compatible osmolyte trehalose, reported to present a synergistic effect on LEAs protective effect [30]. The resulting graphs showed that, when 100 mM trehalose is present, a 1:1 ASR1:CS ratio is sufficient to prevent CS aggregation (Fig 3D). We also noted, that the protective effect of ASR1 is stronger when compared with trehalose alone, resulting in a synergistic effect on chaperone activity. In summary, Zn^2+^ showed no alterations on the ASR1-driven protective effect in any of the conditions assayed. Notably, CD spectra showed that the presence of trehalose did not modify the ASR1 secondary structure either in the presence or absence of Zn^2+^ (S3 Fig). From these data, we can conclude that the partial ASR1 folding induced by Zn^2+^ did not alter ASR1 chaperone behavior and that the synergistic effect of trehalose cannot be attributed to changes on ASR1 folding.

### Following ASR1 structure *in vivo*

#### FRET reporter design

**Fig 4:**
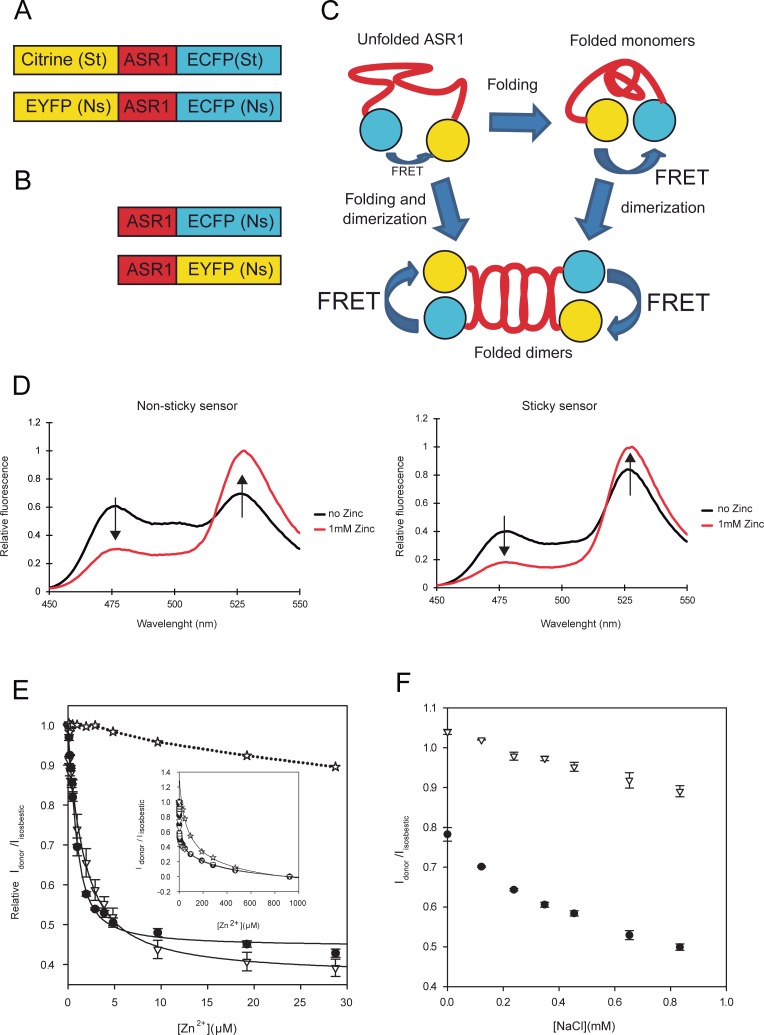
FRET sensor. A) Description of full-length FRET reporters. B) Illustration of single tagged-ASR1 constructs. C) Diagram of possible events that may modify FRET efficiency of the ASR1 sensor. D) Fluorescent spectra of purified sticky and non-sticky reporter proteins in the presence or absence of 1 mM Zn^2+^. E) *In vitro* Zn^2+^ titration (1 μM protein) in low micromolar and nearly millimolar ranges (inset) for sticky (circles), non-sticky (triangles) and equal amounts of both single-tagged proteins (stars). Relative (I_donor_/I_Isosbestic_) ratios are shown. Lines correspond to the best fit to Eq 5 for the low micromolar range and Eq 7 for nearly micromolar range. F) NaCl-induced stress assays in *E*. *coli* cells expressing sticky (circles) or non-sticky probes (triangles). Relative (I_donor_/I_Isosbestic_) ratios as a function of NaCl concentration are shown.

In order to monitor ASR1 structural changes *in vivo*, we designed a fluorescent chimeric ASR1 folding-reporter protein based on FRET. The design was inspired in the CALWY-4 zinc sensor [31] using the well-characterized FRET pair Cerulean—EYFP. As CALWY-4 takes advantage of fluorescent proteins that tend to dimerize in a “sticky” way, we also designed a non-sticky version of the construct by using ECFP and EYFP (Fig 4A and 4B). Taking into account that ASR1 is prone to adopt quaternary structure [11][9], we reasoned that the construct would be able to detect ASR1 homo-dimerization as well as folding (Fig 4C). To distinguish between these two scenarios, we also created single-tagged fusion proteins (fused to non-sticky fluorescent proteins moieties) to precisely assess the effect of dimerization on FRET efficiency (Fig 4B).

#### Testing FRET sensor *in vitro*

In the same way as assayed with the recombinant ASR1 protein in CD experiments, we used Zn^2+^ in order to induce ASR1 folding. The observed spectrum showed a high initial efficiency of FRET for the sticky sensor with an approximately 3-fold increase in efficiency upon incubation with 1 mM ZnCl_2_ (Fig 4D). In the case of the non-sticky version, the protein showed a lower initial FRET efficiency and an increase of approximately 4-fold after the addition of 1mM ZnCl_2_. Even though the non-sticky sensor showed a better dynamic range than the sticky one (4-fold and 3-fold changes, respectively) both of them seems to be useful to report ASR1 structural alterations.

A more detailed study was done by Zn^2+^ titration experiments with the purified recombinant sensors. Relative I_donor_/I_Isosbestic_ values as a function of Zn^2+^concentration showed two binding events (Fig 4E). The first event saturates at a Zn^2+^ concentration similar to that observed in the binding plot obtained by CD (roughly 50 μM). In contrast, the second step saturates at Zn^2+^ concentrations as high as millimolar. Titration experiments were also performed with single-tagged proteins in equimolar amounts. Our data show that the binding event in the low micromolar Zn^2+^ range was absent with the single-tagged proteins, indicating that this first Zn^2+^ binding is not involved in dimer formation. Only the second event was observed with the single-tagged proteins, suggesting that higher Zn^2+^ concentrations promote ASR1 dimerization (Fig 4E inset). These results are in accordance with the behaviour of untagged ASR1, which showed that the protein is a monomer in the lower Zn^2+^ range.

To obtain the stoichiometry for the first Zn^2+^ binding event, we calculated the intersection point between the linear regression comprising the initial rise (0 to 0.2 μM) and the saturation end (9 to 30 μM) of the titration curves for both sensors. In this way, we obtained an intersection point of 1.1 ± 0.1, which confirmed the 1:1 binding model used in CD titration experiments. Using this model, we obtained an equilibrium dissociation constant $K_{d}^{{Zn}^{2+}}$ of (1.31 ± 0.15) μM and (0.45 ± 0.10) μM for non-sticky and sticky reporters, respectively. The presence of the second Zn^2+^ binding event was observed in single-tagged proteins experiments as well. We estimated an average dimerization constant, K_dim_, of (2.6± 0.6) x 10^9^ M^-2^, which enables us to propose a model where an extra Zn^2+^ promotes the formation of a dimer between two ASR1-Zn^2+^ preformed complexes (see Eqs 6 and 7). As expected, the addition of EDTA restored the initial I_donorr_/I_Isobestic_ ratios (S1 Table).

#### FRET sensor responds to saline and osmotic stress *in vivo*

In order to assess if stressful conditions induce ASR1 folding *in vivo*, we expressed the reporter constructs in *Escherichia coli* at different concentrations of NaCl. This salt is known to induce both saline and osmotic stress and, as a consequence of the latter, molecular crowding due to the reduction of cytosol volume [32]. Volume reduction was confirmed by observation of the cells on a confocal microscope before and after the treatment (S4 Fig). Both sticky and non-sticky protein constructs responded to stress by increasing FRET (Fig 4F). Consistently with the observations during protein purification, the sticky sensor showed 5 to 10 times higher expression levels compared to the non-sticky. However, in both cases, the extent of protein expression was enough to allow for a good signal-to-noise ratio. As it has been recently reported that molecular crowding (particularly protein crowding) can affect fluorescent protein performance [33], we followed ASR1 conformational structuring through both I_donor_/I_Isobestic_ and I_aceptor_/I_Isobestic_ ratios. We observed that both ratios responded in the same manner with some differences in the amplitude, especially in the case of the sticky reporter (S5 Fig). When comparing sticky and non-sticky reporters, we found that the first one exhibited a better response (43% change on I_donor_/I_Isobestic_ in comparison to 16%).

## Discussion

We have presented here an exhaustive characterization of ASR1 folding behavior. Overall, ASR1 showed the typical IDP plasticity that allows to easily (ΔG~ 1 kcal/mol) adopt different conformations like α-helix or PII. On one hand, low temperatures, low pH, and high NaCl concentration produced the stabilization of PII structure. On the other hand, macromolecular crowding and Zn^2+^ favored α-helix conformation. This flexibility might be of physiological relevance, as it would allow ASR1 to readily respond to chilling, salinity and drying as it was proposed for other members of the LEA superfamily [12].

Folding upon Zn^2+^ binding has been already reported for other IDPs [24]. In the particular case of ASR1, the addition of Zn^2+^ at a micromolar concentration promoted the protein to stabilize 16% of its polypeptide chain into a α-helix conformation and drastically reduce its hydrodynamic radius. Interestingly, DLS experiments showed two populations that corresponded to the expected radius for natively unfolded (2.95 nm) and globular folded (1.82 nm) protein monomers of 12.7 kDa [28]. Our DLS results differ from previous data [8] reporting an increment in the ASR1 hydrodynamic radius in the presence of Zn^2+^. A very likely explanation is that the above-mentioned work was performed at high concentrations of Zn^2+^ (nearly 1mM) at which ASR1 is already folded and dimerized. In this context, our estimated K_D_s for this first binding (9 μM by CD experiments or 0.5–1 μM by the FRET sensors) are rather higher than the reported constants for known globular zinc-dependent proteins but are in agreement with K_D_s calculated for other IDPs [34]. For instance, GmASR, the soybean ASR1 ortholog, showed two Zn^2+^-binding events as well [35]. The authors proposed that the first Zn^2+^ is bound to GmASR monomer with an affinity similar to that obtained for tomato ASR1 (K_D_ for GmASR = 11± 1 μM).

For ASR1, we found that the second Zn^2+^ binding event induced protein dimerization as inferred from the single-tagged experiments. Interestingly, previous works showed that (HIS)_6_ tags can act as zinc-dependent dimerization domains [36], with a K_dim_ value similar to the observed one for ASR1. Our own control experiments with the (HIS)_6_CLY-6 construct also showed that one event of Zn^2+^binding is able to cause dimerization (S6 Fig). Further assays need to be done to uncover if the “natural (HIS)_5_ tag” of ASR1 leads the protein dimerization event as in the case of (HIS)_6_CLY-6 and its potential relevance in living plant cells under drought stress.

Our data revealed that ASR1 has a rather low affinity towards its specific binding sequence (TGGGCY) when compared with conventional, folded transcription factors. Again, this is not surprising for the case of IDPs, which tend to have lower binding affinities for their ligands or substrates [37]. Interestingly, its DNA-dissociation constant (250 nM) was completely dependent on Zn^2+^. Consistently, a previous report on ASR1-DNA binding showed ASR1 binding to a circular 3-kb dsDNA, with no sequence-specificity, as both monomers and homodimers [9]. Another group proposed that ASR1 binds DNA as a homodimer at high Zn^2+^ concentrations that would promote dimerization [8]. In this framework, fluorescence anisotropy experiments suggested that ASR1 can indeed bind DNA as a monomer in a 1 to 1 ratio. When performing binding assays with a scrambled DNA sequence, we observed a 1 μM dissociation constant. Values of this order of magnitude have been assigned to non-specific electrostatic attractions between transcription factors and DNA [38].

Moving to an *in vivo* setting, the hyperosmotic shock is known to bring about a reduction of cytosolic water. For example, *Escherichia coli* cells lose 40% of cytosolic volume a few seconds after exposure to 1 M NaCl [32]. This decrease in volume leads to an immediate increase in the concentration of all cellular macromolecular compounds, including proteins and ions like Zn^2+^. This, in turn, increases macromolecular crowding within the cell [39]. In this context, a recent work suggested that changes in cell volume may represent an important fine-tuning control of weakly bound protein-protein complexes. For instance, the loss of cell volume and a subsequent increase in intracellular protein concentration could lead to the formation of low-affinity complexes [40]. This scenario is likely to occur not only involving protein-protein interactions but also affecting protein-DNA complexes. Our *in vivo* experiments point out that cell volume reduction (due to saline and osmotic stress) induced ASR1 structuring in the same way as zinc-binding does *in-vitro*. The observed increase in FRET efficiency may be a consequence of ASR1 folding and/or dimerization. Further assays using probes with separated fluorophores would allow us to distinguish between these two possibilities.

Finally, based on our observations and the fact that ASR1 binds DNA in a Zn^2+^-dependent manner, we can conclude that ASR1 may act as a transcription factor when adopting the adequate α-helix conformation. Regarding its chaperone role, here we proved that it is not affected by ASR1 structuring upon addition of Zn^2+^. There is a previous report on a synergistic protective effect between ASR1 and the osmolyte glycine-betaine [13]. Analogously, we revealed synergism of ASR1 combined with the osmolyte trehalose. Such a synergistic effect on chaperone activity does not seem to be due to ASR1 structuring. Rather, our observations support a model in which unstructured domains contribute to chaperone activity [41]. In summary, these results highlight the great plasticity of ASR1 structure that may allow the fine-tuning of its biological roles.

## Supporting information

S1 Fig SDS-PAGE of purified proteins. Purified proteins were loaded into SDS-PAGE gels and stained with Coomassie Brillant Blue or transferred to nitrocellulose membrane and stained with Ponceau Red.
(PDF)

S2 Fig Effect of Zn ^2+^ on ASR1 secondary structure. Comparison of CD spectra of ASR1 (2 μM in Buffer T without Zn ^2+^, with 50 μM Zn ^2+^ and the recovery after the addition of EDTA 100 mM.
(PDF)

S3 Fig Effect of trehalose on ASR1 secondary structure. Comparison of CD spectra of ASR1 (2 μM in Buffer T without or with 50 μM Zn ^2+^) in the absence or presence of 100 mM trehalose.
(PDF)

S4 Fig Relevant data S4 Fig. A) *E*. *coli* cells expressing the non-sticky reporter were observed at a confocal microscope before and after osmotic stress by the addition of 0.83 M NaCl. Settings: Leica Sp8 microscope, Excitation 515 nm, emission 525–600 nm. 1.2 NA water immersion 60x objective and 4X or 10X zoom. Scale is specified for each zoom level. Arrow heads highlight zones were the cytoplasm retracted from the cell wall. B) Series of spectra recording of *E*. *coli* cells expressing the non-sticky showing the raw data used to generate the graph on Fig 4 F. Each curve corresponds to the whole acquired spectra with the specified NaCl concentration. Isosbestic point is marked with an arrow.
(PDF)

S5 Fig Comparison between I_donor_/I_Isobestic_ and I_aceptor_/II_sobestic_ ratios for the FRET sensors in *E*. *coli* cells under NaCl-induced stress. I_donor_/I_Isosbestic_ ratio (left axis, black) and I_aceptor_/I_Isosbestic_ ratio (right axis, red) as a function of NaCl concentration. A) sticky sensor and B) non-sticky sensor. Both ratios responded in the same manner with some differences in the amplitude, especially in the case of the sticky reporter.
(PDF)

S6 Fig Comparison between the FRET sensor and the (HIS)_6_CLY-6 construct. *In vitro* Zn^2+^ titration (1 μM protein) in nearly millimolar ranges for the different constructs employed: sticky sensor (blue circles), non-sticky sensor (empty triangles), equal amounts of both single-tagged proteins (stars) and control experiments with the (HIS)_6_CLY-6 construct (red squares). Relative (I_donor_/I_Isosbestic_) ratios are shown as a function of Zn^2+^concentration. Lines correspond to the best fit to Eq 7.
(PDF)

S1 Table Effect of EDTA addition on I_donor_/I_Isobestic_ values of purified sticky and non-sticky reporter proteins. Table shows how the addition of 5mM EDTA to chelate Zn^2+^, restores the I_donor_/I_Isobestic_ values to the observed prior any Zn^2+^ addition. Mean with its corresponding standard deviation of three independent experiments are shown. The addition of 1mM Zinc and 5mM EDTA were performed sequentially on the same sample.
(PDF)
