# Supplementary figures and images for: Conformational plasticity of the intrinsically disordered protein ASR1 modulates its function as a drought stress-responsive gene
Source: PLoS One. 2018 Aug 23;13(8):e0202808. doi: 10.1371/journal.pone.0202808 (PMC6107238; doi:10.1371/journal.pone.0202808)

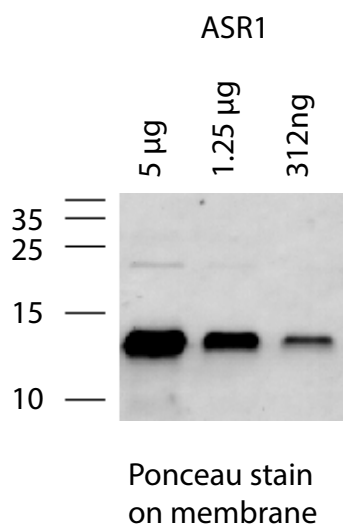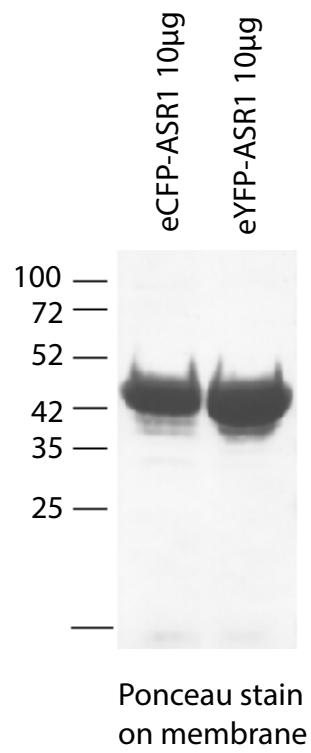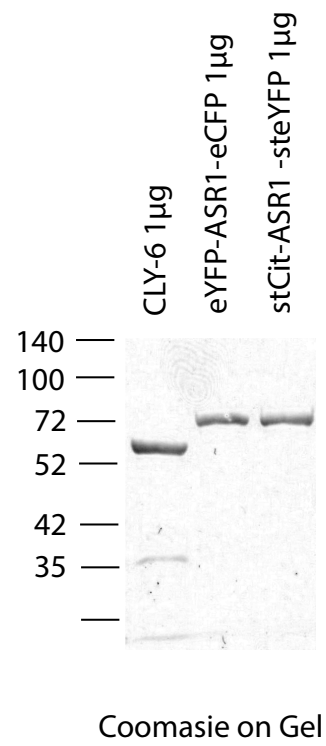

Supplement: S1 Fig — Purified proteins were loaded into SDS-PAGE gels and stained with Coomassie Brillant Blue or transferred to nitrocellulose membrane and stained with Ponceau Red. (PDF) [file pone.0202808.s001.pdf]

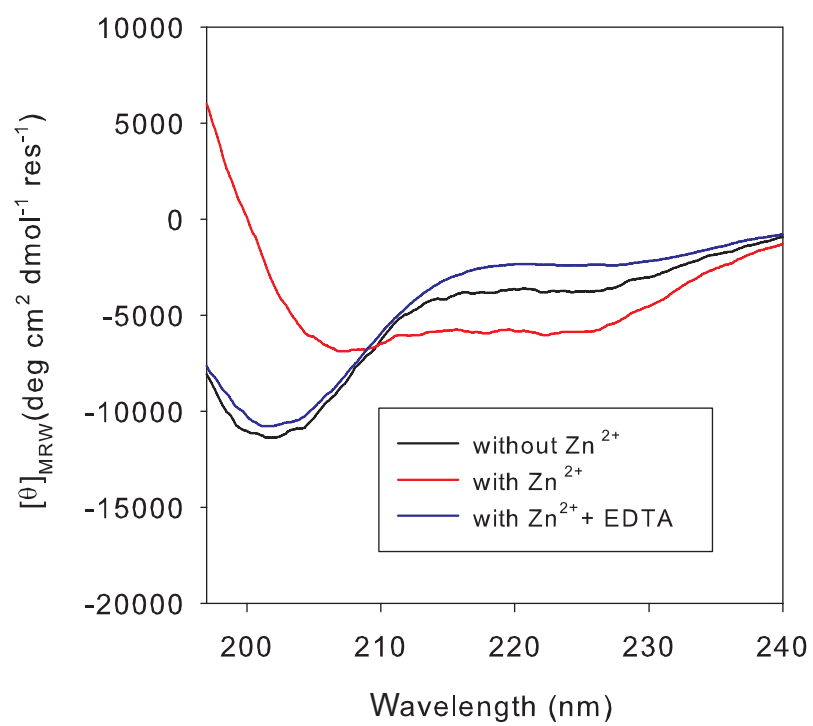

Supplement: S2 Fig — Comparison of CD spectra of ASR1 (2 μM in Buffer T without Zn 2+, with 50 μM Zn 2+ and the recovery after the addition of EDTA 100 mM. (PDF) [file pone.0202808.s002.pdf]

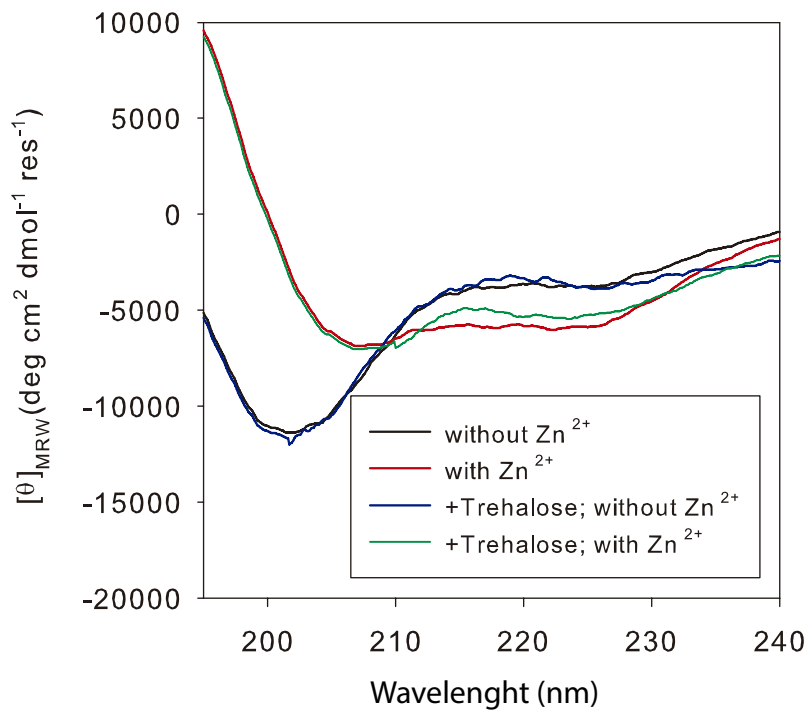

Supplement: S3 Fig — Comparison of CD spectra of ASR1 (2 μM in Buffer T without or with 50 μM Zn 2+) in the absence or presence of 100 mM trehalose. (PDF) [file pone.0202808.s003.pdf]

A

60x, 4X zoom

60x, 10X zoom

0 M NaCl

1 M NaCl

0 M NaCl

1 M NaCl

eYFP channel

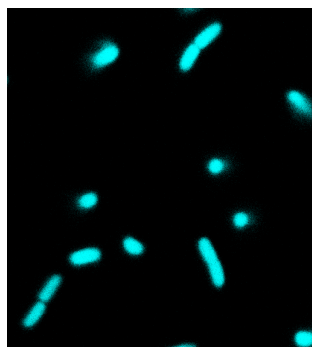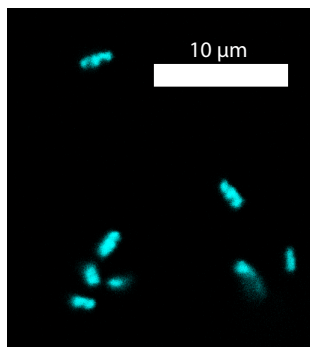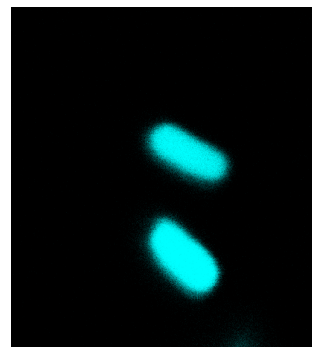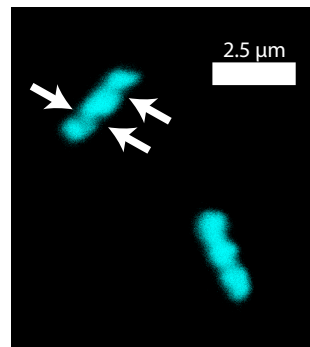

Transmitted

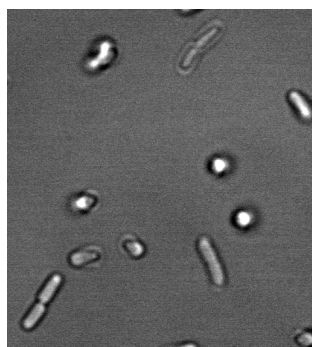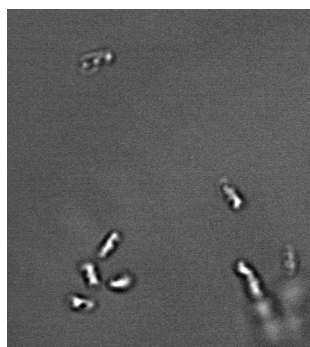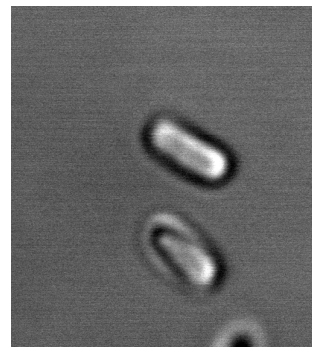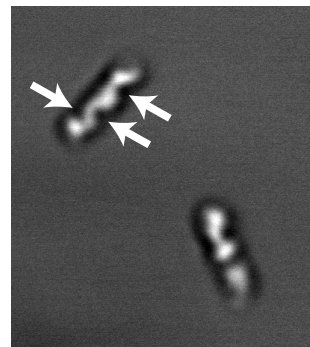

B

Non-sticky reporter

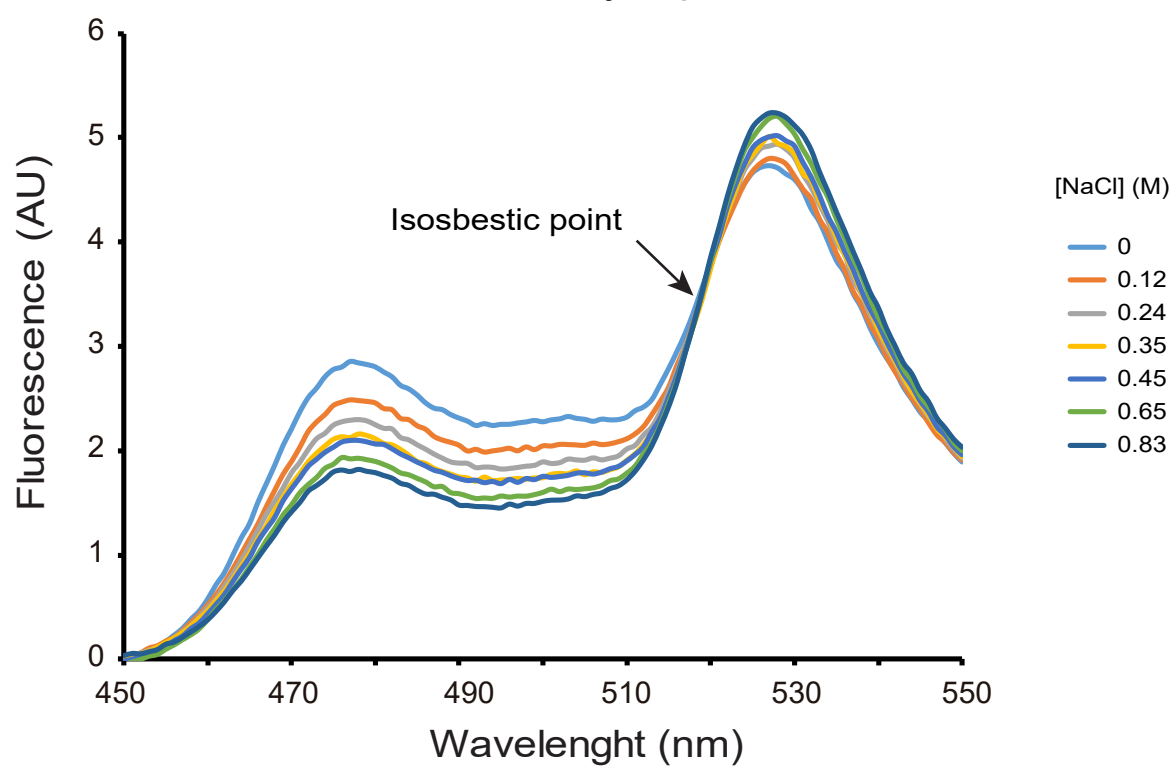

Supplement: S4 Fig — A) E. coli cells expressing the non-sticky reporter were observed at a confocal microscope before and after osmotic stress by the addition of 0.83 M NaCl. Settings: Leica Sp8 microscope, Excitation 515 nm, emission 525–600 nm. 1.2 NA water immersion 60x objective and 4X or 10X zoom. Scale is specified for each zoom level. Arrow heads highlight zones were the cytoplasm retracted from the cell wall. B) Series of spectra recording of E. coli cells expressing the non-sticky showing the raw data used to generate the graph on Fig 4 F. Each curve corresponds to the whole acquired spectra with the specified NaCl concentration. Isosbestic point is marked with an arrow. (PDF) [file pone.0202808.s004.pdf]

**A**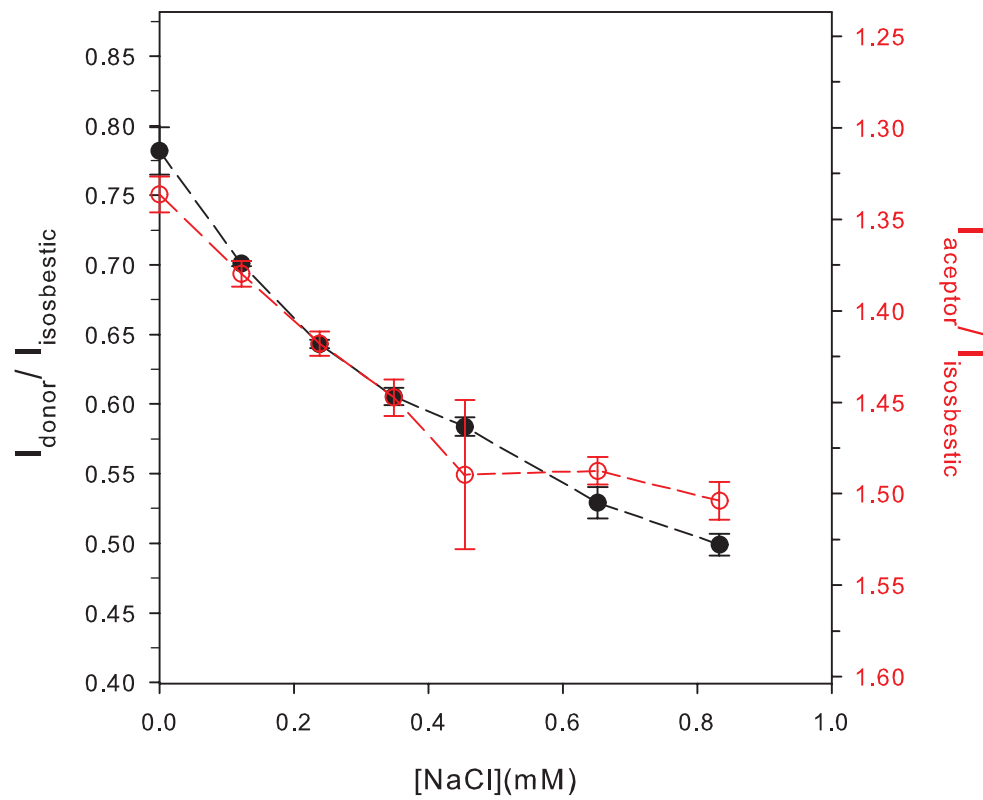**B**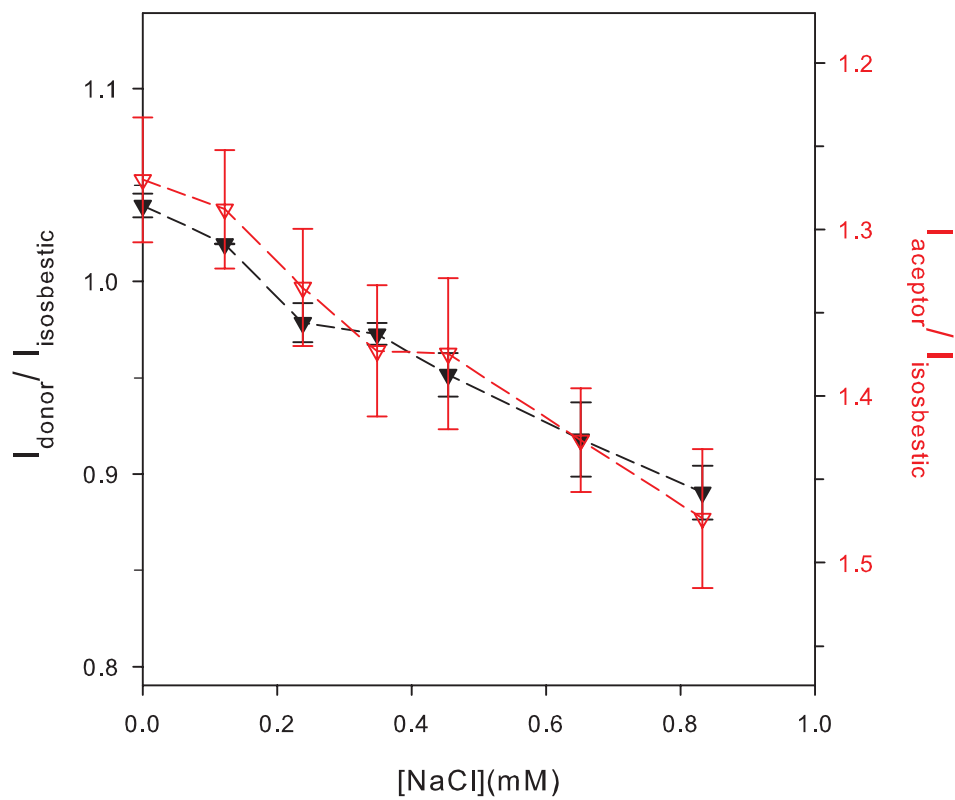

Supplement: S5 Fig — Idonor/IIsosbestic ratio (left axis, black) and Iaceptor/IIsosbestic ratio (right axis, red) as a function of NaCl concentration. A) sticky sensor and B) non-sticky sensor. Both ratios responded in the same manner with some differences in the amplitude, especially in the case of the sticky reporter. (PDF) [file pone.0202808.s005.pdf]

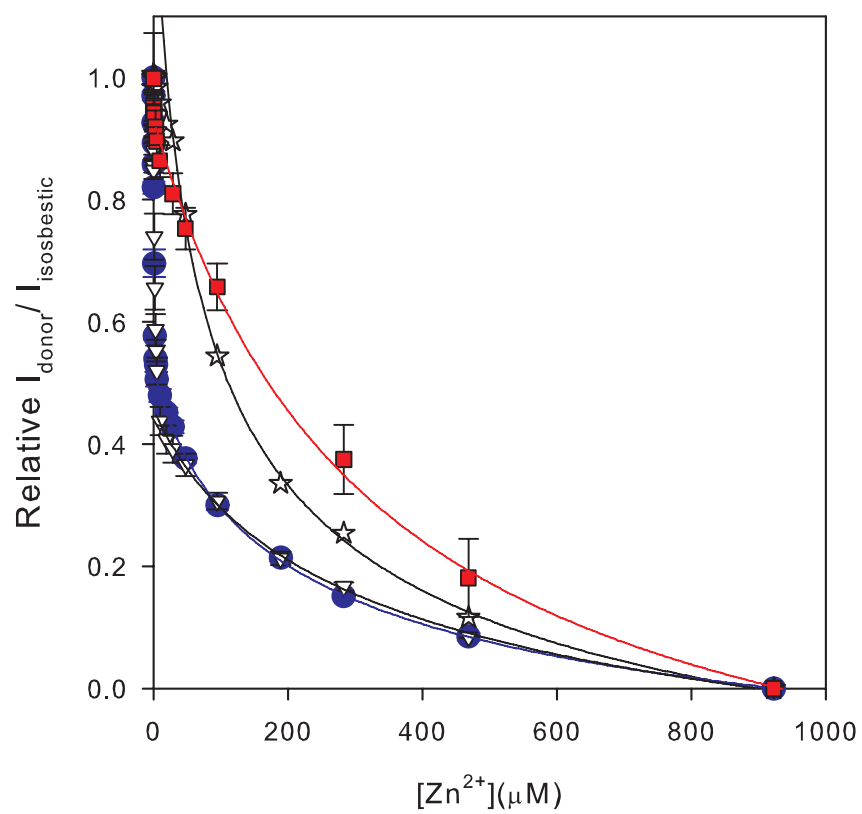

Supplement: S6 Fig — In vitro Zn2+ titration (1 μM protein) in nearly millimolar ranges for the different constructs employed: sticky sensor (blue circles), non-sticky sensor (empty triangles), equal amounts of both single-tagged proteins (stars) and control experiments with the (HIS)6CLY-6 construct (red squares). Relative (Idonor/IIsosbestic) ratios are shown as a function of Zn2+concentration. Lines correspond to the best fit to Eq 7. (PDF) [file pone.0202808.s006.pdf]
