# Supplementary material for: Conformational plasticity of the intrinsically disordered protein ASR1 modulates its function as a drought stress-responsive gene
Source: PLoS One. 2018 Aug 23;13(8):e0202808. doi: 10.1371/journal.pone.0202808 (PMC6107238; doi:10.1371/journal.pone.0202808)
Supplement: S1 Table — Table shows how the addition of 5mM EDTA to chelate Zn2+, restores the Idonor/IIsobestic values to the observed prior any Zn2+ addition. Mean with its corresponding standard deviation of three independent experiments are shown. The addition of 1mM Zinc and 5mM EDTA were performed sequentially on the same sample. (PDF) [file pone.0202808.s007.pdf]

	No Zinc		1mM Zinc		1mM Zn + EDTA	
Non-sticky reporter	1.357	±0.069	0.581	±0.037	1.308	±0.018
Sticky reporter	0.637	±0.022	0.302	±0.002	0.562	±0.003
